# Supplementary material for: Super-resolution modularity analysis shows polyhedral caveolin-1 oligomers combine to form scaffolds and caveolae
Source: Sci Rep. 2019 Jul 8;9:9888. doi: 10.1038/s41598-019-46174-z (PMC6614455; doi:10.1038/s41598-019-46174-z)
Supplement: Supplementary file 1 — Supplementary Legends and Figure [file 41598_2019_46174_MOESM1_ESM.pdf]

Super-resolution modularity analysis shows polyhedral caveolin-1 oligomers combine to form scaffolds and caveolae

Ismail M. Khater[1], **Qian Liu**[2], **Keng C. Chou**[2], **Ghassan Hamarneh**[1*], **Ivan Robert Nabi**[3*#]

SUPPLEMENTAL FIGURE LEGEND

Supp. Figure S1. Additional features for Cav1 blobs. Cav1 blob biosignatures in addition to those presented in Fig. 2C are shown for the matched groups from PC3, CAVIN1/PTRF-transfected PC3 (PC3-PTRF), and HeLa cells.

SUPPLEMENTAL VIDEO LEGEND

Supp. Video S1. Animated rotating structures of S1A scaffolds (H3), S1B scaffolds (H4), S2 scaffolds (H1), and caveolae (H2) blobs in HeLa cells. The blob's localizations, connections between localizations (edges), and modules (for S1B, S2 scaffolds, and caveolae) are shown from different angles with rotation. The video illustrates Cav1 domain formation as described in Figure 5. Rotating blobs in 3D enhance visualization of the internal structure of the blobs. For example, the hollow nature of caveolae is clear when rotating the views.
